# Radiocarbon signatures of carbon phases exported by Swiss rivers in the Anthropocene

**DOI:** 10.1098/rsta.2022.0326

**Published:** 2023-11-27

**Authors:** Timo M. Y. Rhyner, Lisa Bröder, Margot E. White, Benedict V. A. Mittelbach, Alexander Brunmayr, Frank Hagedorn, Florian R. Storck, Lucas Passera, Negar Haghipour, Juerg Zobrist, Timothy I. Eglinton

**Affiliations:** ^1^ Geological Institute, ETH Zürich, 8092 Zürich, Switzerland; ^2^ Department of Physics, Laboratory of Ion Beam Physics,8093 Zürich, Switzerland; ^3^ Hydrology Division, Federal Office for the Environment, 3003 Bern, Switzerland; ^4^ Swiss Federal Institute for Forest, Snow, and Landscape Research, 8903 Birmensdorf, Switzerland; ^5^ Department of Physics, Imperial College London,SW7 2AZ London, UK; ^6^ Emeritus Scientist, Swiss Federal Institute of Aquatic Science and Technology, 8600 Dübendorf, Switzerland

**Keywords:** Switzerland, radiocarbon, rivers, global carbon cycle, Anthropocene, land-to-ocean-aquatic-continuum

## Abstract

Lateral carbon transport through the land-to-ocean-aquatic-continuum (LOAC) represents a key component of the global carbon cycle. This LOAC involves complex processes, many of which are prone to anthropogenic perturbation, yet the influence of natural and human-induced drivers remains poorly constrained. This study examines the radiocarbon (^14^C) signatures of particulate and dissolved organic carbon (POC, DOC) and dissolved inorganic carbon (DIC) transported by Swiss rivers to assess controls on sources and cycling of carbon within their watersheds. Twenty-one rivers were selected and sampled during high-flow conditions in summer 2021, a year of exceptionally high rainfall. Δ^14^C values of POC range from −446‰ to −158‰, while corresponding ranges of Δ^14^C values for DOC and DIC are −377‰ to −43‰ and −301‰ to −40‰, respectively, indicating the prevalence of pre-aged carbon. Region-specific agricultural practices seem to have an influential effect on all three carbon phases in rivers draining the Swiss Plateau. Based on Multivariate Regression Analysis, mean basin elevation correlated negatively with Δ^14^C values of all three carbon phases. These contrasts between alpine terrain and the lowlands reflect the importance of overriding ecoregional controls on riverine carbon dynamics within Switzerland, despite high spatial variability in catchment properties.

This article is part of the Theo Murphy meeting issue 'Radiocarbon in the Anthropocene'.

## 1. Introduction

Fossil CO_2_ emissions since the era of industrialization together with substantial changes in land use are major contributors to climate change. More frequent extreme weather events such as heatwaves, droughts and storms are superimposed on gradual changes in temperature and hydroclimate [1–4]. Changes to the hydrological cycle affect the global carbon cycle, and human activities alone have increased global erosion rates and transport of sediments to an extent that exceeds Earth's natural processes [5–7]. As the effects of anthropogenically driven climate change become a tangible reality, a deeper understanding of how these changes are linked to perturbations of Earth's carbon cycle has grown in importance. Therefore, there is a need to constrain and quantify changes to the hydrological and carbon cycles at a broad range of scales, including regional to global, and to distinguish anthropogenic perturbations from natural baseline variability [1,8–10].

Lateral carbon fluxes represent important vectors that influence the fate of carbon taken up from the atmosphere by the terrestrial biosphere, transporting carbon from one reservoir to another and redistributing it along the land-to-ocean-aquatic-continuum (LOAC) [9]. These lateral processes are particularly prone to anthropogenic perturbations due to human activities on the land surface, yet remain poorly constrained [9,11,12]. In this regard, rivers serve as sentinels of carbon cycle change and natural integrators of processes occurring within their watersheds, mobilizing and transforming carbon during its movement from source to sink [11]. Our understanding of the role of rivers has evolved from the concept of a simple pipeline to a more reactive system interacting with its surroundings [13]. In general, riverine carbon dynamics vary by catchment characteristics, such as lithology, geomorphology, climate and hydrology [8,14–18]. Extreme hydrologic events (e.g. heavy rainfall or snow/ice melting events) can exert a large influence on carbon mobilization by shunting carbon from terrestrial interfaces to streams [19]. Human activities disrupt both landscapes and the natural functioning of river systems, impacting them in myriad ways including nutrient inputs from fertilizers and sewage discharge, and construction of dams for hydroelectric power and freshwater storage [12,20–23]. However, the relative importance of the different natural drivers modulating fluvial carbon export, and their susceptibility to anthropogenic perturbation, remains uncertain.

Radiocarbon (^14^C) serves as a powerful tool to constrain carbon sources and dynamics on a range of spatial and temporal scales [24–27]. Fluvially transported carbon may exhibit sharply differing radiocarbon characteristics due to varying inputs of modern biospheric carbon from vegetation, pre-aged carbon stored in soils and ancient petrogenic carbon derived from rock weathering [28–33]. Besides source apportionment, radiocarbon activity also enables the investigation of the controls on ecosystem-scale carbon turnover times [15,34]. Radiocarbon ages of dissolved organic carbon (DOC) in rivers tend to be relatively young (enriched in ^14^C, i.e. higher Δ^14^C values), reflecting mostly fresh biospheric inputs [8,28,35], whereas radiocarbon ages for particulate organic carbon (POC) generally exhibit a broader range in ages from old (depleted in ^14^C, i.e. lower Δ^14^C values) in the headwaters to young in the lowland rivers [8,28]. The latter may reflect dilution or remineralization processes whereby ancient (radiocarbon-free) petrogenic carbon is superseded by carbon from fresh vegetation both from land and in-stream production during downstream transport [8,17,28]. The radiocarbon characteristics of riverine dissolved inorganic carbon (DIC) are influenced by gas exchange with the atmosphere, organic matter remineralization processes and bedrock chemical weathering pathways. With respect to the latter, riverine DIC can exhibit higher Δ^14^C values (modern ages), representing silicate weathering by carbonic acid derived from precipitation or biospheric carbon respiration, or lower Δ^14^C values (older ages) stemming from carbonic acid weathering of carbonate rocks. Contributions of radiocarbon-dead or fossil radiocarbon may also result of weathering of carbonates by sulfuric acid or oxidation of petrogenic OC [8,28,36]. Consequently, riverine DIC generally exhibits lower Δ^14^C values in mountainous headwaters. Depending on the weathering regime these processes can either represent a source or a sink for atmospheric CO_2_ and reflect a significant carbon input to upland streams [37,38].

Prior radiocarbon studies in rivers have mainly focused on major river systems due to their global importance in regulating freshwater and materials fluxes to the ocean (e.g. [28,39]). More recent work has highlighted the collective role of small, mountainous river systems draining active continental margins (e.g. Taiwan) as globally important vectors for sediment and carbon translocation and export [40,41]. Other studies have emphasized the importance of smaller headwater streams and inland waters along the LOAC both as important sources of CO_2_ to the atmosphere and as integral components of the global carbon cycle [9,42]. While many prior studies tend to focus on either the inorganic or organic phase of carbon due to contrasting (e.g. geochemical or ecological) perspectives [8,18,32,40,43,44], studies examining all three C phases (DIC, DOC, POC), especially using radiocarbon [20], remain sparse. However, given the intimate relationships between these different phases, simultaneous characterization of the three carbon phases may help to elucidate carbon dynamics within the freshwater aquatic continuum [9].

**Figure 1.**
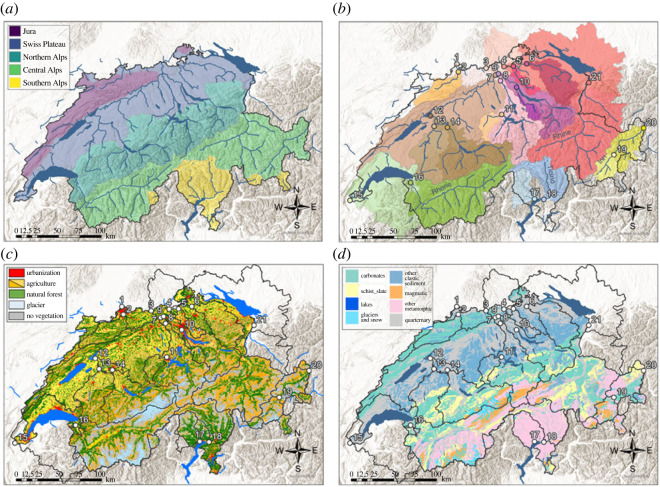
(*a*) Map of Switzerland and its five ecoregions. Major rivers and lakes are indicated in blue. (*b*) Map of Switzerland with 21 river sampling stations indicated as numbered points. Different colours indicate different watersheds. Station locations are coloured according to the colour of the corresponding watershed. Major rivers and lakes are indicated in blue. (*c*) Map of Switzerland where different colours indicate different types of land use. Yellow is intensive agriculture and orange is alpine agriculture. The watershed area of each station is indicated with a dark grey line. Major lakes are indicated in blue. (*d*) Map of Switzerland where different colours indicate different types of underlying bedrock lithology.

Switzerland, particularly in its alpine regions, is experiencing environmental and ecosystem change at a faster pace than most regions of the world [45,46]. This change is manifested in rapidly retreating glaciers, decreasing snow and permafrost coverage, alpine greening, and increasing intensity and frequency of extreme rain events and droughts [34,45–48]. Over the past four decades, river water temperatures increased by 0.8–1.3°C, while water discharge remained largely unchanged. For the major three drainage basins in Switzerland (Rhine, Rhone and Ticino), there was a small but statistically significant increase in DIC concentrations over this time interval [49]. This suggests increased DIC inputs from bedrock weathering, belowground respiration and/or soil OM remineralization in aquatic systems, all of which are potentially accelerated by increasing temperatures due to global warming. These observations provide motivation for the present study, which investigates the radiocarbon-isotopic characteristics of POC, DOC and DIC currently transported by Swiss rivers. We examine a suite of 21 rivers draining the five different ecoregions of Switzerland (Jura, Plateau, Northern-, Central- and Southern Alps). These ecoregions host a broad range of drainage basins that are characterized by sharp contrasts in elevation, geomorphology, bedrock lithology, climatic properties such as temperature, hydrological and cryospheric characteristics and associated ecosystems they support, but also anthropogenic influences such as river channelization, dams and land use [14,50]. Because the major drainage basins of Switzerland map onto these different ecoregions (figure 1*a* and electronic supplementary material, table S5), they lend themselves to the assessment of regional-scale controls on the amount and composition of carbon exported by the different river systems and provide a window into the impact of future scenarios of climate change on Swiss landscapes. Thus, following a space-for-time approach, assessment of responses to global warming can, for example, be addressed through investigation of the Southern Alps ecoregion (Ticino rivers), which exhibits higher temperature anomalies than other parts of Switzerland. Given the complex mosaic of drainage basin characteristics, we assess whether any overarching factors emerge that control the amount, source and composition of carbon that the rivers export. In this context, radiocarbon serves as both a tracer and metric of carbon inputs and processes that control its turnover and fate. This assessment is based on measurement of samples collected at each of the river stations during the summer season with the goal of capturing the period of highest water and sediment discharge. In order to isolate major drivers, we conduct a Multivariate Regression Analysis, incorporating *in situ* water quality measurements and a long-term hydrological dataset stemming from the National Long-Term Surveillance of Rivers (NADUF) programme, combined with land cover characteristics of the watersheds. Ultimately, we seek to assess the sources and pathways of carbon within the aquatic continuum as a function of differences in catchment characteristics that may inform on future changes of the C cycle in response to direct and indirect anthropogenic perturbation.

## 2. Methods

### (a) Study site and sampling stations

Switzerland hosts a plethora of river systems, including the headwater streams feeding the four major central European rivers—the Rhine, Rhone, Ticino and Inn (the latter two representing headwaters of the Po and Danube, respectively). Lakes Constance, Geneva and Maggiore are the receiving basins for the Upper Rhine, Upper Rhone and Ticino rivers, respectively. Rivers in Switzerland are exceptionally well monitored regarding their discharge and water chemistry, primarily as a result of the NADUF programme, which has been reporting ongoing changes in Swiss rivers and their watersheds for 50 years [14,49–53]. A total of 19 sampling stations were chosen to align with those of the NADUF programme (figure 1*b*). In addition to the NADUF-stations, two additional rivers—the Sihl River (at Brunau, station 10) and the Maggia River (at Locarno, station 17)—were chosen, the former (and its headwaters) being the focus of prior in-depth investigations [17,43].

Land-use within the different river basins differs considerably (figure 1*c*). The northern part of Switzerland, the Swiss Plateau ecoregion, is heavily anthropogenically impacted and is primarily characterized by agricultural land use (cropland) and urban settlements. By contrast, while also anthropogenically perturbed, alpine regions are covered by natural and managed forests, pastures and mostly unproductive areas due to steep terrain and barren land surfaces [45] (figure 1*c*). Pressures related to agricultural land use (e.g. inputs of fertilizers, manures, etc.) thus follow a general gradient of increasing anthropogenic perturbation from south to north, although extensive practices of agriculture are common in the southern part of Switzerland (canton Ticino) as well, and patches of urbanization are also found in some mountainous valleys.

Underlying bedrock can be an important factor influencing the age, amount and compositional characteristics of both POC and DIC found in rivers. Switzerland is covered by a variety of lithologies, which for this study are categorized into six groups (figure 1*d*). Magmatic rocks are commonly found in the central Alps, where the headwaters of most major Swiss rivers are located. Metamorphic rocks including gneiss and schists are commonly found in the south and east of Switzerland. Carbonate rocks, predominantly limestone and dolomite, form large parts of the pre-Alps and the Jura mountains in the north of the country. DIC is especially affected by the chemical weathering of carbonate rocks, where they contribute old, i.e. radiocarbon-dead, carbon to the DIC pool [36]. Sedimentary rocks, such as slates and shales, mostly of marine origin, are found in the uppermost Rhine valley and the lower Engadin valley. Riverine POC can be affected by the erosion of petrogenic OC from such sedimentary rocks, which contain ancient (i.e. radiocarbon-free) OC (kerogen) [54]. Siliciclastic sediments and unconsolidated quaternary sediments are found in the Swiss Plateau region, where the latter is also found along the major river valleys in all regions. Streamflow seasonality in Swiss river systems is foremost governed by precipitation and snowmelt [49,51,53]. Additionally, hydropower facilities significantly modulate the discharge of Swiss rivers. Generally, the maximum discharge occurs during the spring and summer seasons, with alpine rivers exhibiting stronger seasonality as a consequence of snow and ice melt [14,49]. Compared with a rapid response in peak flow for alpine rivers, monitoring stations located downstream of major lakes respond rather slowly and with lower amplitude to flood events [14,49,51].

### (b) Sampling methodology

With the exception of a few cases (table 1), river waters were sampled between 8 May and 25 August 2021. We focus on periods of high discharge since previous studies have shown that most of the carbon in rivers is transported during high flow conditions [14,43,49]. For this study, we therefore only report data corresponding to the sampling date with the highest discharge on the premise that this represents the dominant radiocarbon signatures of carbon phases for each river (table 1). Surface water samples (less than 0.5 m) were collected from the middle of the river channel (usually from bridges) with a pre-rinsed metal bucket. Using a custom-made filtration system (capacity 2.5 l), the river water was pressure-filtered through a 90 mm diameter and 0.2 µm pore size polyether sulfone (PES) filter using a bicycle pump (max. press., 2 bar) [40,55]. For POC determination, the PES filters (Whatman Millipore) were placed into 40 ml pre-combusted glass vials or aluminium foil pouches and then stored frozen (−20°C) prior to analysis. Before sample preparation, PES-filters were freeze-dried and then wetted with MilliQ-water, then the vials were placed into an ultrasonic bath and vortexed to efficiently release the sediment particles. Sediment suspensions were then freeze-dried before subsampling. Only for one station (station 8 at Mellingen), a pre-combusted glass fibre filter (GF/F, Whatman Millipore, 0.7 µm pore size) was used with a pre-combusted glass filtration set-up and a vacuum-pump. After freeze drying the GFF-filter, several 4 mm circles were punched out and placed into silver boats before fumigation.

**Table 1.** Study site information and ^14^C results. Study side information included is mean basin area, mean basin elevation, average monthly discharge of all available data for the same sampling month, sampling date and corresponding discharge. The ratio of discharge during sampling compared to monthly average of all available FOEN data of the same month is shown (Ratio). ^14^C-values for particulate (POC) and dissolved (DOC) organic carbon as well as dissolved inorganic carbon (DIC) are reported as Δ^14^C-values.

			mean watershed	monthly		discharge		Δ^14^C-values (‰)		
station	name	area (km^2^)	elevation (m)	discharge (m^3 ^s ^−1^)	date	(m^3^s^−1^)	ratio	PO^14^C	DO^14^C	DI^14^C
1	Rhine-Weil	36 472	1055	1457 (1891–2018)	22 July 2021	2426	1.7	−59	−133	−138
2	Birs-Münchenstein	14 718	733	11 (1917–2018)	22 July 2021	29.5	2.7	−107	−150	−120
3	Rhine-Laufenburg	34 040	1078	1522 (1980–1985)	21 July 2021	2400	1.6	−124	−77	−161
4	Rhine-Rekingen	14 767	1134	670 (1904–2018)	28 June 2021	614.3	0.9	−122	−88	−174
5	Glatt-Rheinsfelden	417	506	8.75 (1976–2018)	28 June 2021	7.4	0.8	−142	−158	−160
6	Thur-Andelfingen	1696	773	49.9 (1904–2018)	19 July 2021	129	2.6	−106	−43	−138
7	Aare-Brugg	11 726	1003	376 (1935–2018)	9 Aug 2021	737	2.0	−92	−143	−92
8	Reuss-Mellingen	3385	1258	191 (1935–2018)	2 Aug 2021	385.7	2.0	−158	−53	−177
9	Limmat-Gebenstorf	2393	1066	114 (1951–2018)	9 Aug 2021	179	1.6	−193	−103	−188
10	Sihl-Brunau	342	1047	7.83 (1938–2018)	14 July 2021	85.8	11.0	−122	−186	−245
11	Kleine Emme-Littau	478.3	1058	11.3 (1985–2018)	12 July 2021	21.1	1.9	−116	−48	−163
12	Aare-Hagneck	5104	1368	263 (1984–2015)	7 July 2021	435	1.7	−172	−118	−144^b^
13	Sanne-Gümmenen	1881	1135	15.2 (1981–2018)	7 July 2021	107.5	7.1	−114	−67	−156^b^
14	Aare-BernSchönau	2941	1591	211 (1935–2018)	7 July 2021	331	1.6	−198	−172	−172^b^
15	Rhone-Chancy	10 323	1570	512 (1935–2018)	6 July 2021	430	0.8	−258	−165	−41^a,b^
16	Rhone-Porte du Scex	5244	2124	351 (1935–2018)	6 July 2021	285	0.8	−218	−377	−291^b^
17	Maggia-Locarno	926	1534	13.8 (1985–2018)	20 May 2021	10.8	0.8	−92	−208	−131
18	Ticino-Riazzino	1613	1643	148 (1999–2005)	20 May 2021	77.8	0.5	−150	−258	−301
19	Inn-S-chanf	618	2460	38.4 (1998–2021)	25 Aug 2021	25.5	0.7	−132	−235	−208
20	Inn-Martina	1941	2342	71.9 (1970–2018)	8 May 2021	81.4	1.1	−446	−164	−126
21	Rhine-Diepoldsau	6119	1771	448 (1983–2018)	25 June 2021	466	1.0	−252	−278	−264

^a^Five values for DI^14^C represent exceptions that were resampled during the month of October 2021, outside of the main sampling period.

^b^Unusually small sample (7 µgC; 1.88% error).

DOC samples (filtrate) were collected in pre-combusted amber glass bottles (250 ml) and stored in the freezer at −20°C until measurement. DIC was sampled separately using a 50 ml syringe and 0.2 µm PES Sterivex-filter (Sigma Aldrich) to remove particulate matter. The filtrate was sampled into 12 ml exetainer vials pre-poisoned with mercuric chloride (HgCl_2_) to eliminate bacterial activity and subsequently stored cold (4°C) in the dark prior to measurement.

### (c) Analytical methods

Radiocarbon analyses were conducted using a MICADAS (Mini Carbon Dating System) Accelerator Mass spectrometer (AMS) [56] equipped with a Gas Interfaces System (GIS) and CO_2_-accepting ion source at the Laboratory for Ion Beam Physics (LIP) in the Department of Physics at the ETH Zurich. Before being wrapped into tin capsules, approximately 25 mg of suspended matter (POC) was weighed into silver capsules and then fumigated over HCl (37%) vapours (65°C, 72 h) to remove inorganic carbon and subsequently neutralized by exposure to sodium hydroxide pellets (65°C, 72 h). Radiocarbon is measured using the on-line elemental analyzer (EA)-AMS system [57]. For DO^14^C, around 30 ml of pre-filtered water was freeze-dried. Then phosphoric acid (85%) was added to remove DIC. A wet chemical oxidation (WCO) method is applied, which is based on using aqueous persulfate oxidant to oxidize the DOC, and subsequent purging and radiocarbon analysis of released CO_2_ through an automated headspace sampler coupled to the MICADAS [58]. All the DOC samples have been corrected with the constant contamination (Mc = 0.98 ± 0.45, F14C = 0.39 ± 0.08) method according to [59]. DI^14^C-samples have been purged with helium in order to remove atmospheric CO_2_ from the DIC-sample. Then 250 µl of phosphoric acid (85%) is added into the samples and the CO_2_ in the headspace liberated from conversion of DIC in the vial introduced to the gas interface system (GIS) [60] and measured with the gas ion source of MICADAS. For all the DIC samples, C1 (IAEA) was used as a blank material and C2 (IAEA) was used as a secondary reference material. All ^14^C-values are reported as F^14^C-values according to [61] which are then converted to Δ^14^C-values $(\Delta^{14}\text{C}=[\text{Fm}*e^{\lambda (1950-Y_{c})}-1]*1000),$ where *λ* is the inverse of the true mean-life of radiocarbon and *Y_c_* is the year of collection. The Δ^14^C is age corrected to account for decay that took place between collection and the time of measurement so that two measurements of the same sample made years apart will produce the same calculated Δ^14^C result. Absolute percentage error was less than 1.5% for all samples with the exception of one indicated with a superscript "a" (^a^) in table 1. Exact percentage error is reported in the electronic supplementary material, tables S7 and S8.

### (d) Statistical analysis

In order to examine potential relationships between response and control variables an Ordinary Least Squares (OLS) Multivariate Regression Analysis (MRA) was performed according to standardized procedures [62] and plotted as a Redundancy Analysis (RDA). We standardized all values in order to facilitate comparisons between various parameters of different units. In short, for all data we subtracted the mean and divided by the standard deviation for each variable. All statistical analysis was conducted using R-studio version 13 with the vegan package. The snapshot dataset of bulk F^14^C-values for the three carbon phases (POC, DOC and DIC) are selected as response variables, whereas different watershed parameters—land cover, lithology, topography—and climatic, hydrologic as well as anthropogenic variables were selected as the control variables. A digital elevation model (DHM25, 25 m grid) is used as the basis for the relief analysis (calculation of the partial catchment areas and flow paths) using the software ArcGIS version 10 (ESRI 2011). The methodology is based on the use of Swiss official governmental digital data, where the control variables about land cover were calculated as the basin average according to the ‘Areal Coverage Data Set 2020’, provided by Federal Office for the Environment (FOEN^1^). For lithology coverage, ‘Origin of Rocks 500 Data Set’ was used (provided by FOEN, see footnote 1). All other control variables such as information about catchment topography, climate and hydrology were compiled from the dataset of the National Long-Term Surveillance of River Program (NADUF^2^).

## 3. Results

### (a) River discharge and water chemistry

The sampling was conducted during summer 2021, a year of extreme rain events resulting in episodes of exceptionally high discharge that had not been witnessed in decades. For example, at station 1 (Rhine at Weil), the most downstream station of the Rhine in Switzerland with a watershed area covering the majority of the Swiss territory, average discharge measured by the NADUF-Program during summer 2021 was 2426 m^3^ s^−1^. This discharge value has not been observed since the summer of 1999 (3217 m^3^ s^−1^) (NADUF). For most of the sampling stations, it was possible to capture the peak of discharge during the year of 2021 (table 1), and thus our snapshot samples primarily reflect high discharge events.

The last stations of the Rhine River, station 1 at Weil and 3 at Laufenburg, exhibited the highest discharge at the time of sampling of 2426 and 2400 m^3^ s^−1^, respectively, followed by station 7 (Aare River at Brugg) and station 21 (Rhine at Diepoldsau) with 737 and 466 m^3^ s^−1^, respectively (table 1). Station 5 (Glatt River at Rheinsfelden), by contrast, showed the lowest discharge of 7.4 m^3^ s^−1^ during our sampling in 2021. Average river water temperature of our sampling set was 16.4 ± 3.8°C (*n* = 21). The maximum temperature value of 22.5°C was at station 5 (Glatt River at Rheinsfelden), whereas the minimum of 9.2°C was at station 20 (Inn River at Martina). The average river water pH value of our sampling set was 8.3 ± 0.14. The maximum pH value (8.5) was at station 20 (Inn River at Martina), whereas the minimum (7.9) was at station 17 (Maggia River at Locarno; electronic supplementary material, table S3).

### (b) Radiocarbon signatures

**Figure 2.**
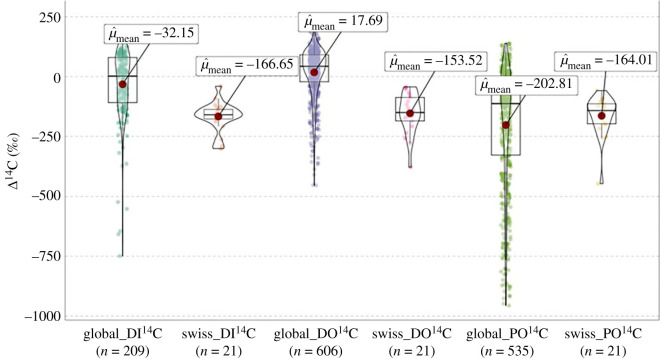
Violin plots of radiocarbon phases (DI^14^C, DO^14^C, PO^14^C) with boxplots showing median values of Δ^14^C. Red dots indicate the mean values, whereas the thick line within the boxplot indicates the average value. Swiss ^14^C phases are compared against the global compilation from [28]. Radiocarbon values are expressed in Δ^14^C notation. Coloured dots indicate the range and variability of single measurements.

The average Δ^14^C value of POC was −164.3 ± 86.2‰, while average Δ^14^C values for DOC and DIC were −153.5 ± 84.7‰ and −166.6 ± 60.9‰, respectively (*n* = 21 for each, figure 2), indicating the presence of pre-aged carbon in all three pools. Δ^14^C measurements for all samples showed an absolute percentage error of less than 1.5%, with the exception of one sample indicated with a superscript "a" (^a^), which is subject to greater uncertainty (1.88% = ±18‰, table 1). No samples from the present study yielded Δ^14^C values corresponding to modern (post-bomb) age (i.e. Δ^14^C > 0‰). The lowest Δ^14^C value for POC (−446‰) was measured at station 20 (Inn River at Martina) in the Engadin Valley, which displayed the lowest value (oldest ^14^C age) of all three carbon pools (*n* = 63; table 1), while the highest PO^14^C value of −58‰ was found at station 1 (Rhine River at Weil), the most downstream site of the Rhine River. DOC Δ^14^C values ranged from −377‰ at station 16 (Rhone River at Porte du Scex) to −43‰ at station 6 (Thur River at Andelfingen). DIC Δ^14^C values ranged from −301‰ at station 18 (Ticino River at Riazzino) to −41‰ at station 15 (Rhone River at Chancy), the latter being the highest value (youngest ^14^C age) out of all three carbon pools. The amplitude of variability in DIC Δ^14^C values was smaller than for both DOC and POC, with POC Δ^14^C exhibiting the highest amplitude (figure 2). In general, Δ^14^C values of all three carbon phases follow a similar pattern. Stations on rivers draining the alpine region in southern Switzerland generally show the lowest Δ^14^C values, whereas those in the northern part of Switzerland draining the Swiss Plateau are higher. In addition to the natural climate, geological and ecological contrasts between northern and southern regions that may influence Δ^14^C values, anthropogenic influences (e.g. population density, agriculture) also follow this general pattern. A comparison with the global Δ^14^C compilation dataset by Marwick *et al*. [28] shows that Swiss Rivers export carbon characterized by lower Δ^14^C values than the global average (figure 2), particularly for DIC and DOC.

### (c) Univariate regression analysis

**Figure 3.**
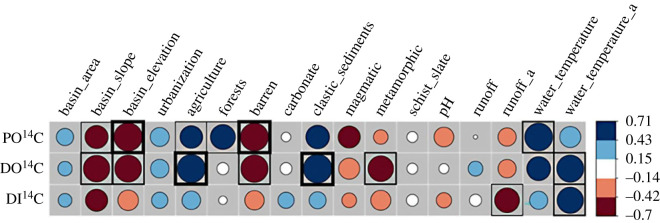
Matrix of Pearson correlation coefficients (*r*-values) between land use, lithology, topography and water parameters (controls) and radiocarbon values (responses). Circle sizes and colours correspond to the strength of the correlation. Correlations that are significant at the *p* = 0.05, *p* = 0.01 and *p* = 0.001 level are outlined with a thin, regular and thick black border, respectively. Variables with an ‘a’ at the end indicate annual averages from the past decade extracted from the NADUF dataset (2012–2020).

The Pearson correlation plot showed that all three carbon pools (PO^14^C, DO^14^C, DI^14^C) correlate in a similar way regarding their relationships with different watershed and fluvial parameters (figure 3). Annual river temperature extracted from the NADUF dataset of the past decade (2012–2020) showed significant negative correlation with DO^14^C and DI^14^C (*R*^2^ = 0.41; *p*-value: <0.005 and *R*^2^ = 0.37; *p*-value: <0.01, respectively), but not with PO^14^C. Runoff and discharge values corresponding to the sampling day showed no correlation, whereas annual average runoff values extrapolated from the NADUF dataset of the last decade (2012–2020) had a negative correlation with DI^14^C (*R*^2^ = 0.30; *p*-value: <0.05; figure 3). Mean annual basin precipitation (1971–2020) did not exhibit any significant relationships (electronic supplementary material, table S6).

Regarding topographic features within the catchment basin, mean basin slope showed a significant but weak negative correlation with Δ^14^C signatures of PO^14^C and DO^14^C (*R*^2^ = 0.22; *p*-value: <0.05; *R*^2^ = 0.37; *p*-value: <0.01, respectively), but not to DI^14^C. Similarly, mean basin elevation shows a strong significant negative correlation with both PO^14^C and DO^14^C (*R*^2^ = 0.49; *p*-value: <0.001, and *R*^2^ = 0.39; *p*-value: <0.005, respectively), but not with DI^14^C. No correlation was evident between basin area and any of the radiocarbon pools (figure 3). With respect to land-use type, mean basin cover of agricultural fields, including farmland, alpine agriculture and pastural land, exhibits a significant positive correlation to DO^14^C (*R*^2^ = 0.47; *p*-value: <0.001). By contrast, there is a negative correlation between barren areas with PO^14^C and DO^14^C (*R*^2^ = 0.46; *p*-value: <0.001, and *R*^2^ = 0.38; *p*-value: <0.005, respectively, figure 3). For PO^14^C only, there was a positive correlation with forest coverage within the catchment (*R*^2^ = 0.30; *p*-value: <0.01). Population density did not show any significant relationships with Δ^14^C signatures (electronic supplementary material, table S6). Concerning bedrock lithology, average basin cover of clastic sediments exhibits a significant positive correlation with DO^14^C (*R*^2^ = 0.50; *p*-value: <0.001), although it should be noted that clastic sediments strongly correlate with agricultural land-use (*R*^2^ = 0.63; *p*-value: <0.001). There is also negative correlation of metamorphic rocks and DO^14^C (*R*^2^ = 0.32; *p*-value: <0.05).

A significant positive correlation was found between DO^14^C and DI^14^C (*R*^2^ = 0.31; *p*-value: <0.01; electronic supplementary material, table S6). Besides this, there were no other significant correlations between Δ^14^C-values of different carbon phases.

### (d) Multivariate regression analysis

**Figure 4.**
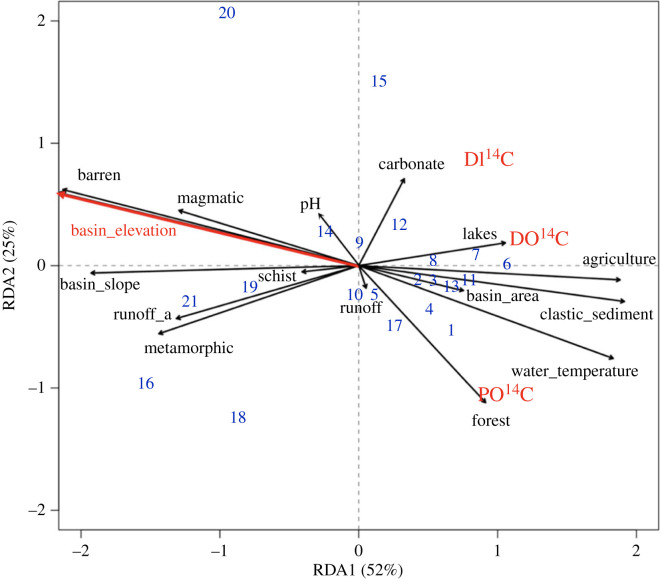
RDA plot showing the RDA1 and RDA2 canonical axes. Environmental control variable loadings are plotted as black arrows where significant vectors are illustrated in red (basin_elevation), PO^14^C, DO^14^C and DI^14^C response variable loadings are plotted in red, and individual sampling stations (table 1) are plotted as blue numbers. Variables annotated with an ‘_a’ correspond to annual average values of the past decade (2012–2020).

The outcome of the RDA reveals that two orthogonal axes explain a combined 77% of the total sample variation (figure 4). For the first axis (RDA1: 52%; *p*-value: <0.001), parameters such as barren areas, agriculture, catchment slope and elevation are loaded. The second axis (RDA2: 25%; *p*-value: <0.1) loads closely with average cover of carbonate rock, forest and pH. As the only significant environmental variable, mean basin elevation seems to exert a major influence on all three carbon phases (*p*-value: <0.001). The adjusted *R*^2^-value of this model is 0.32 (electronic supplementary material, table S6).

## 4. Discussion

### (a) Representativeness of high discharge conditions

It is important to note that the samples used for this investigation were taken as a snapshot at high flow. Such data obtained during anomalous hydrological conditions require careful interpretation and proper awareness of its limitations as well as possible biased perspectives it may create. While further work is clearly needed to assess whether similar relationships between radiocarbon signatures and drainage basin properties hold for other discharge conditions, we believe these initial findings are nevertheless informative.

Previous studies have shown that there is a positive relationship between runoff and organic carbon concentration in Swiss rivers [14,43,49,51,63]. Other studies of similar river systems outside Switzerland support this, especially during the wet season or with the onset of the spring thaw [64–71]. With respect to radiocarbon, according to recent studies of a sub-alpine Swiss catchment, riverine POC radiocarbon signatures are more variable under low flow conditions but tend to cluster around higher values during above-average river discharge conditions [17,43]. This implies that samples collected during high flow conditions are likely to be more representative, while also accounting for the majority of carbon export.

The elevated discharge during our sampling meets these conditions. Above-average carbon fluxes and uniform radiocarbon signatures likely reflect carbon supply predominantly via surface runoff and soil erosion that would contribute to higher Δ^14^C values [17,43,66,69,70]. Thus, our snapshot dataset may disproportionately reflect specific endmembers and lead to a radiocarbon signature that is biased towards higher Δ^14^C values with the magnitude of this bias dependent upon catchment characteristics. Nevertheless, from a carbon cycle perspective, and with a view to isolating the overarching factors controlling fluvial carbon export, above-average discharge conditions should represent the predominant state of a river system. To ensure sound comparison between river systems, and considering practical constraints, we argue that the focus on above-average discharge conditions is warranted.

### (b) Controls on riverine radiocarbon

River systems are highly complex, as are the controls on riverine carbon dynamics [14,17,51,53]. The identification of drivers of carbon signatures in river systems thus remains challenging, particularly in diverse and heterogeneous watersheds such as those within Switzerland where multiple factors overlap spatially. Compared with the global riverine radiocarbon data compilation, our radiocarbon measurements yield generally lower values ([28]; figure 2). This difference may be partly due to the different sampling years of both datasets and resulting manifestation of bomb-derived radiocarbon in carbon pools, especially in comparison with samples collected closer to its peak in 1963.

### (c) Particulate organic carbon

Eglinton *et al*. [15] showed from radiocarbon measurements of source-specific organic compounds that climate modulation of soil carbon turnover times dictates riverine terrestrial biospheric PO^14^C values at the global scale. Accordingly, slower soil carbon turnover in alpine settings could account for lower Δ^14^C values of riverine POC for higher elevation stations. Our study does not refute this conclusion at the national scale across a pronounced climatic gradient given that Δ^14^C values of riverine POC are lower in alpine settings, which have lower soil organic carbon (SOC) stocks than lowland soils [50]. Given the marked differences in climatic regimes among the five different ecoregions (figure 1*a* and electronic supplementary material, table S5), Switzerland may therefore echo these global-scale patterns. In agreement with this, temperature, precipitation and moisture have been identified as major drivers governing SOC stocks in Switzerland [50], while other authors have argued that at the regional scale, physiochemical properties such as soil pH, moisture and mineralogy override the controls of climatic regimes on SOC dynamics in surface soils [72]. Alternatively, increased soil loss in response to extreme rainfall on Swiss grasslands has been observed to generally occur during July and September, coinciding with our sampling campaign [73,74], and this could lead to increased inputs of POC with lower Δ^14^C values from the erosion of deeper mineral soils. Additional measurements, including stable isotopic (^13^C) analysis of POC, are needed to confirm potential contributions of SOC to the river system. Petrogenic carbon inputs from bedrock weathering and erosion of sedimentary rock mobilized by rain events, freeze–thaw processes or glacier retreat represent additional potential sources of radiocarbon-depleted or radiocarbon-free carbon to rivers draining the Alpine terrain, independent of direct influences of soil-derived carbon inputs and soil turnover times. In particular, the terrain above the tree line in the Alps comprised incompletely weathered sedimentary material [75], and steeper slopes erode deeper soil layers where such contributions have been observed to manifest themselves in lower Δ^14^C values of POC [76]. The metamorphic sedimentary ‘Bündnerschiefer’ lithology, which outcrops in various watersheds (e.g. Inn and Upper Rhine), could contribute to lower Δ^14^C values, particularly at station 20 of the Inn River at Martina in the lower Engadin, which is surrounded by this type of schist and exhibited the lowest Δ^14^C value for POC within our dataset. In these alpine regions, further efforts are thus needed to distinguish petrogenic POC inputs of fossil age from those derived from pre-aged soil OC.

Downstream in the Swiss Plateau, where Δ^14^C values are generally higher, PO^14^C shows a positive correlation to average basin cover of forests, suggesting the latter comprise a source of fresher biospheric C (figure 3). Storm-facilitated export of carbon has been linked with modern biospheric sources [67,70], where especially during summer months an enhanced contribution of vegetation-derived POC has been reported in a Swiss sub-alpine catchment [17]. The positive correlation of agricultural land use and PO^14^C as well as DO^14^C strongly suggests that the endmembers of higher Δ^14^C values for DOC and POC might have a similar origin or mode of supply (figure 3). Overall, in addition to climatic differences, the stark contrast between Swiss alpine terrain versus lowland Swiss Plateau also reflects a gradient in anthropogenic pressures (e.g. urbanization and agriculture), contributions from upstream restricted carbon sources, as well as dilution and transformation processes by increased soil, plant biomass inputs and in-stream aquatic productivity [14,17,49].

### (d) Dissolved organic carbon

Based on univariate regression analysis, PO^14^C showed strong negative correlations with several parameters which were also significant for DO^14^C, suggesting some common sources with low Δ^14^C values from headwater streams. However, there was no significant correlation between the Δ^14^C values of these two carbon phases (electronic supplementary material, table S6), implying that they are also influenced by other drivers. As previous studies have observed, lower DOC Δ^14^C values can derive from various sources such as shallow and deep soil layers, groundwater inputs, karst systems and natural springs, but also from organisms incorporating inorganic carbon from bedrock weathering [32,33]. Input from a single source with lower Δ^14^C value for POC (e.g. erosion of rock or pre-aged soils), in contrast to multiple sources and input processes of lower Δ^14^C DOC (e.g. soil leaching, in-stream productivity, groundwater and glacial meltwater inputs), might explain such an overlapping trend, while also reconciling the lack of relationship between PO^14^C and DO^14^C.

DOC in lowland rivers is often derived from fresh vegetation or leaching of surface soils, thus ubiquitously high in Δ^14^C values [28]. Agricultural farmlands could enhance this input of young carbon through fresh crops or manure. Studies have pointed out that excessive inputs of manure from pastural activities can lead to eutrophication, markedly increasing in-stream productivity [77,78], which in turn can lead to higher Δ^14^C values for DOC. It has also been argued that agricultural practices could supply DOC of lower Δ^14^C values to rivers through the exhumation of deeper soil layers, such as following conversion of forests to cropland (which increases soil erosion due to weaker soil stability with less extensive root systems; [79,80]). However, compared with the low Δ^14^C signatures emanating from upland regions of Swiss rivers, the magnitude of change in DOC Δ^14^C values would instead correspond to higher Δ^14^C values [79,81,82] given that Swiss riverine DOC generally exhibits relatively low Δ^14^C values compared with the global average [28,32]. It has been argued that in-stream productivity of major lowland rivers, where flow velocities and suspended sediment concentrations diminish, are significant contributors of riverine organic carbon [33,83]. Moreover, Chen *et al*. [83] recently suggested that even in mountainous rivers in-stream production and transformation of carbon exerts a significant influence on riverine DO^14^C dynamics. Riverine in-stream transformations can be separated into biotic (e.g. primary and secondary production) or abiotic (e.g. absorption, desorption, photo-oxidation, dissolution) processes, which are each strongly controlled by river water temperature and residence time [33,35,83]. The significant positive relationship between DO^14^C and the long-term average of annual river water temperature from the NADUF dataset supports the assumption of significant in-stream productivity. However, such long-term trends in river water temperature almost certainly also reflect similar trends in air and land surface temperature, with attendant changes in terrestrial productivity and soil DOC dynamics. Enhanced DOC leaching from soils was observed particularly when increased precipitation was coupled with increasing soil temperatures, as highlighted by an *in situ* soil warming study as well as a recent laboratory study [84,85]. Consequently, it remains challenging to attribute the links in river DO^14^C solely to aquatic productivity, and further information (e.g. stable carbon isotope as well as other geochemical data) would help to distinguish allochthonous from autochthonous sources.

### (e) Dissolved inorganic carbon

Of the three carbon phases, DI^14^C exhibits the least significant univariate relationships to watershed variables. This renders it difficult to pinpoint drivers, especially given the complex nature of weathering-related interactions between the lithosphere and atmosphere. Swiss rivers are oversaturated with respect to atmospheric CO_2_ primarily because of weathering of carbonate lithologies. This results in a net outgassing of CO_2_ [49]. By contrast, increased runoff from snow and ice melt can lead to undersaturation of DIC in rivers and consequently to atmospheric CO_2_ uptake [49]. Invasion of atmospheric CO_2_ with high Δ^14^C values into rivers may dilute DIC of low Δ^14^C values derived from chemical weathering of carbon-containing bedrock. Thus, the identification of drivers is further complicated depending on the degree of oversaturation or undersaturation of the river with respect to DIC.

We found no significant correlation between DI^14^C and carbonate lithology or any other broad type of lithological category included in our analysis. The argument that DI^14^C is primarily governed by the combination of kinetics, foremost temperature or organic matter respiration and the interaction with weathering processes rather than the presence of different types of lithology or land use alone could explain this lack of correlation with watershed variables. In contrast to the organic carbon phases, DI^14^C did exhibit a significant negative correlation with average runoff of the past 9 years (2012–2020). Increased precipitation leading to enhanced soil DIC leaching was observed for an *in situ* field experiment and recent laboratory studies [84,85]. Our finding that DI^14^C and average runoff of the past decades (1971–2020) showed a significant correlation may also suggest a link with the small but significant increase in DIC concentration during the past four decades in three major Swiss river systems (Rhine, Rhone and Ticino) [49]. Over the same period, carbonate lithology-related parameters such as alkalinity, total hardness, Ca and Mg have increased by up to 10% [49]. This might suggest the importance of a combined effect in amplifying runoff and carbonate weathering, increasing the supply of radiocarbon-depleted DIC. Degassing of DIC (as CO_2_) from rivers to the atmosphere, coupled with the long-term increases of DIC concentrations observed in major Swiss rivers [49], could thus comprise a significant positive feedback to climate change. Further measurements are necessary to deconvolute signatures and apportion specific contributions from different sources of DIC [36,49].

In our study, we note that DIC Δ^14^C values are higher in the Swiss Plateau (table 1). DI^14^C is positively correlated with DO^14^C, which could reflect DOC remineralization or in-stream production of DOC (electronic supplementary material, table S6). Coupled effects of precipitation and warming [85] and microbial decomposition accelerated by increasing OC inputs due to agricultural practices are possible explanations for such an observation. Comparing sampling sites located in low elevation with high elevation, it becomes evident that decreasing contributions of upstream restricted carbon sources, along with dilution and transformation processes by increasing biospheric inputs might explain this general pattern [14,17,49]. These higher DIC Δ^14^C values in the Swiss Plateau compared with the Swiss Alps possibly originate from enhanced soil organic matter respiration and DIC leaching and could thus override the DI^14^C signatures of ‘alpine characteristic’ further downstream. Additionally, the oversaturation of Swiss rivers with respect to DIC could lead to the outgassing of headwater sourced CO_2_ during downstream transport, while subsequent atmospheric CO_2_ uptake via aquatic autotrophy or DIC emanating from more modern sources further downstream may dilute signals from bedrock weathering and related processes [49]. Nevertheless, considering the degree of change in Δ^14^C values of the two other carbon pools (POC, DOC), the difference in Δ^14^C values for DIC from headwaters to downstream is relatively small (table 1).

### (f) Region-specific controls on carbon isotopic signatures

The concurrence of diverse land cover characteristics within different Swiss river watersheds may lead to the influence of specific controls on radiocarbon signatures cancelling one another out. For example, with respect to POC, the lowest Δ^14^C value (−446‰) was found at station 20 (Inn River at Martina), where carbonaceous schists outcrop extensively in the vicinity of the sampling station (figure 1*d*). Although not in proximity of the sampling site, station 10 (Sihl River at Brunau) and station 13 (Saane River at Gümmenen), both have a higher extent of schists outcropping within their catchments (31% and 21%, respectively, compared with 8% for station 20). Yet their corresponding POC Δ^14^C values (−122‰ and −113‰, respectively) are higher than for station 20 at Martina. One explanation might be that station 10 and 13 have a twofold higher percentage agricultural land cover (44.8% and 54.5%, respectively, compared with 24%; electronic supplementary material, table S1) and a fivefold to eightfold higher cover of pastural farmland (28.9% and 19%, respectively, compared with 3.45%; electronic supplementary material, table S2). Even though erosion of organic-rich schists is known to deliver OC of fossil age, fresh organic carbon inputs from pastural activities might counteract this low POC Δ^14^C signature. Furthermore, when we compare the three most agriculturally dominated catchments (station 6, 11 and 13 at Andelfingen, Littau and Gümmenen, respectively), we observe similar Δ^14^C patterns among all three carbon phases, suggesting an overriding influence of agricultural practices on the carbon pool. Moreover, stations 2, 6, 10, 11 and 13, which have high percentage cover of pastural land (electronic supplementary material, table S2), show a similar range in Δ^14^C values for POC (*ca* −110‰; table 1). This indicates that particularly pastural practices may exert a strong influence on riverine PO^14^C signatures, as also stated in previous studies [86].

Similar counteracting effects may also play a role in masking climatic versus anthropogenic controls on Δ^14^C signatures of DOC. For example, Δ^14^C values for DOC from stations 16 (Rhone at Porte du Scex) and 18 (Ticino at Riazzino) are among the lowest observed for the 21 rivers investigated (−377‰ and −258‰, respectively; table 1). Retreating glaciers in the European Alps are known to release DOC of low Δ^14^C value [87]. This may serve as an explanation for the observation at station 16 given its catchment has by far the highest percentage of glacier and snow cover (15.8%). However, station 18 (Ticino River at Riazzino) accounts for only *ca* 1% of glacier cover (electronic supplementary material, table S2), indicating that there must be an alternative source of DOC with low Δ^14^C values. The Canton of Ticino in the southern Alps, where stations 17 and 18 are located, is known for its warm Mediterranean climate, extensive forests and well-developed soils with high carbon stocks (electronic supplementary material, tables S2 and S4; [50]). This region is also known for its forest fires that produce recalcitrant pyrogenic (black) carbon residues that can accumulate in soils [88], potentially serving as an explanation for lower DOC Δ^14^C values at station 18. Overall, as previously discussed for POC, Δ^14^C values of DOC at the agriculturally impacted stations 6, 11 and 13 are consistently high, ranging from −43‰ to −67‰ (table 1). This again highlights the strong influence of agriculture on Δ^14^C signatures [86]. Flocculation and in-stream transformation of DOC into POC, degradation of POC into DOC, or increased aquatic productivity due to nutrient input might serve as explanations for this observation.

DI^14^C signatures among the 21 rivers exhibited significant spatial variability but did not show any significant correlation with land cover (figure 3). Station 21 (Rhine in Diepoldsau), 16 (Rhone at Porte du Scex) and 18 (Ticino at Riazzino) exhibit the lowest DIC Δ^14^C values (−267‰, −290‰ and −301‰, respectively; table 1). While weathering of carbonate bedrock could be a source of DIC with low Δ^14^C values in the upper Rhine at station 21, this lithology is not predominant in the latter two catchments, where metamorphic rocks predominate instead (32% and 72%, respectively; electronic supplementary material, table S1). Interestingly, DOC ^14^C for these three stations also exhibit low Δ^14^C values (table 1). This might indicate that DIC Δ^14^C values are dependent on the Δ^14^C signature of DOC that undergoes remineralization. For example, it is remarkable how similar all three Δ^14^C values of POC, DOC and DIC are for station 21 (table 1), which suggests well mixed waters in the Rhine River contributing to homogeneous radiocarbon signatures of carbon phases. However, the Δ^14^C value of DIC at station 16 is much higher than that of DOC, whereas the opposite is the case for station 18, contradicting this well mixed behaviour observed at station 21. The agricultural practice of liming could also serve as a further source of DIC with low Δ^14^C value [89,90], although this remains speculative as we lack information on such practices in Switzerland. We note again that Δ^14^C values for DIC at the three most agriculturally impacted stations 6, 11 and 13 are within a similar range from −138‰ to −177‰ (table 1), suggesting that this mode of land-use may modulate riverine DI^14^C signatures.

## 5. Conclusion

A radiocarbon survey of POC, DOC and DIC from 21 Swiss rivers sampled under high flow conditions in spring/summer 2021 reveals a general contrast between watersheds draining the Swiss Alps with lower Δ^14^C values and those on the Swiss Plateau with higher Δ^14^C values. A RDA showing that mean basin elevation is negatively correlated with Δ^14^C values of riverine C phases underlines this general observation. This may reflect either preferential removal of ^14^C-depleted (old, i.e. low Δ^14^C) carbon and/or replacement or dilution by ^14^C-enriched (young, i.e. high Δ^14^C) carbon from in-stream processes or additional inputs along the riverine continuum. Higher flow conditions during the sampling period may have led to a stronger contrast between the Swiss Alps and Swiss Plateau, but higher overall fluxes imply that signatures under these hydrological conditions should be representative of overall discharge. Controls on carbon dynamics within specific catchments, however, are more complex given the interplay between multiple variables (e.g. elevation, barren surfaces, slope, agriculture, lithology), with counteracting factors likely contributing to a lack of correlation among variables and measured radiocarbon signatures. Region-specific factors such as carbonaceous schists and glaciers seem to play a role in delivering radiocarbon-depleted carbon. But foremost, on the Swiss Plateau, agricultural land use (namely pastural activities) exerts a strong influence on radiocarbon signatures showing positive correlation to all three carbon phases, implying a strong anthropogenic imprint on Swiss riverine carbon export.

## Data Availability

The data are provided in the electronic supplementary material [91].

## Footnotes

1 https://data.geo.admin.ch/ch.swisstopo.geologie-geotechnik-gk500-genese and https://www.bfs.admin.ch/bfs/de/home/dienstleistungen/geostat/geodaten-bundesstatistik/boden-nutzung-bedeckung-eignung/arealstatistik-schweiz.assetdetail.20104753.html.

2 https://opendata.eawag.ch/group/naduf-national-long-term-surveillance-of-swiss-rivers.
